# The Importance of Recovery in Resistance Training Microcycle Construction

**DOI:** 10.5114/jhk/186659

**Published:** 2024-04-15

**Authors:** Colby A. Sousa, Michael C. Zourdos, Adam G. Storey, Eric R. Helms

**Affiliations:** 1 Sport Performance Research Institute New Zealand (SPRINZ), Auckland University of Technology, Auckland, New Zealand.; 2 Exercise Science and Health Promotion, Muscle Physiology Laboratory, Florida Atlantic University, Boca Raton, United States.

**Keywords:** fatigue, strength, hypertrophy, monitoring

## Abstract

Systemic resistance training aims to enhance performance by balancing stress, fatigue and recovery. While fatigue is expected, insufficient recovery may temporarily impair performance. The aim of this review was to examine evidence regarding manipulation of resistance training variables on subsequent effects on recovery and performance. PubMed, Medline, SPORTDiscus, Scopus and CINAHL were searched. Only studies that investigated recovery between resistance training sessions were selected, with a total of 24 articles included for review. Training to failure may lengthen recovery times, potentially impairing performance; however, it may be suitable if implemented strategically ensuring adequate recovery between sessions of similar exercises or muscle groups. Higher volumes may increase recovery demands, especially when paired with training to failure, however, with wide variation in individual responses, it is suggested to start with lower volume, monitor recovery, and gradually increase training volume if appropriate. Exercises emphasising the lower body, multi-joint movements, greater muscle recruitment, eccentric contractions, and/or the lengthened position may require longer recovery times. Adjusting volume and frequency of these exercises can affect recovery demands depending on the goals and training logistics. Daily undulating programming may maximise performance on priority sessions while maintaining purposeful and productive easy days. For example, active recovery in the form of training opposing muscle groups, light aerobic cardio, or low-volume power-type training may improve recovery and potentially elicit a post activation potentiation priming effect compared to passive recovery. However, it is possible that training cessation may be adequate for allowing sufficient recovery prior to sessions of importance.

## Introduction

The goal of systemic resistance training (RT) is to improve performance; however, to accomplish this goal a training program must obtain the appropriate balance of stress, fatigue, and recovery (Bird, 2013), which are all interrelated. Fatigue, referred to as physical and/or mental exhaustion causing a deterioration in performance (Plisk and Stone, 2003), is expected from training; however, inadequate recovery may lead to a temporary reduction in force (Brown et al., 1997), decreased performance (Burt and Twist, 2011), and an increased risk of injury (Cheung et al., 2003). Recovery is as a two-stage process involving the reduction of fatigue and adapting to imposed training demands. Specifically, an individual should at least be able to return to baseline performance or potentially, beyond that (Sands et al., 2007).

Trainers and practitioners have the potential to help even highly experienced athletes improve their resistance training performance if they carefully balance their training stress and stimulus (Helms et al., 2020). This starts with having a systematic approach to training through specific and purposeful manipulation of training variables such as volume, loads, and proximity to failure commonly referred to as periodisation (Fleck, 1999); however, there has been discussion about proper use of terminology. Specifically, a clear distinction between programming and periodisation is needed as they represent different aspects of the program design and may cause confusion if used incorrectly (Hammert et al., 2021; Hornsby et al., 2020). Distinct from periodisation, which refers to longer term changes, programming refers primarily to session-to-session or within microcycle changes to training variables, which have a more acute impact on training stress and stimulus. When focusing on recovery, attention is mainly given between sessions within a week of training (i.e., a microcycle) to enhance acute performance with the hope of eliciting further adaptation accumulated from multiple weeks of training (i.e., a mesocycle). These daily changes within a training program are often referred to as daily or weekly undulating programming (D/WUP). DUP approaches are often favoured as they have exhibited a greater degree of muscular strength development compared with linear periodisation (Rhea et al., 2002) in trained individuals. When looking at the effects of periodisation for enhancing muscle hypertrophy, undulating and linear models appear to be equally effective (Evans, 2019); however, studies have not been conducted in trained individuals.

Practically, when constructing a microcycle, considering the amount of recovery required between sessions may influence the placement of certain sessions throughout the week. For example, if there is a session that is of high priority (i.e., heavy single repetitions, technical lifts requiring high focus, high volume sessions, or a combination of these), allowing for enough time to recover prior to this session might enhance performance of this high priority session. This could mean altering the traditional order of DUP training from hypertrophy, strength, then power (HSP) to hypertrophy, power, then strength (HPS), to allow for recovery during a power session following a hypertrophy session, therefore leading to greater training volume and total repetitions in the high priority strength session (Zourdos et al., 2016). This is only one such an example where adjusting microcycle construction led to enhanced recovery, yet no reviews have discussed this topic in depth to facilitate better programming decisions by coaches and trainers.

Therefore, the primary aim of this review was to examine current evidence regarding the influence of microcycle construction factors (e.g., proximity to failure, allocation of training volume, single session difficulty) on recovery between resistance training sessions. Primarily, research examining the manipulation of resistance training variables and the subsequent effects on recovery were addressed with additional insight into the role of programming to allow for appropriate recovery and long-term adaptation.

## Methods

To inform this narrative review, PubMed, Medline, SPORTDiscus, Scopus and CINAHL electronic databases were searched online in addition to further hand searching of the reference lists of articles found. In the Scopus database, the subject area was limited to “medicine” and “health professions” with only “articles”, “reviews”, and “articles in press” included for search parameters. The search string: (resistance OR strength OR weight) AND training AND recover* AND athlet* was used for initial selection of manuscripts, limiting database results to peer reviewed studies of human subjects in English.

After obtaining all manuscript records, initial screening included: (i) screening for duplicates; (ii) screening titles for relevance; (iii) screening the abstracts for relevance; (iv) screening the full paper for inclusion criteria; and, (v) reviewing the references of the included papers to find any additional relevant publications that were not included previously. For a study to be included the researchers must have investigated recovery between resistance training sessions within a microcycle.

Due to the variation in methods across studies, this review is presented in a narrative format with the intention of providing an overview of the current literature, new perspectives on training program construction, and direction for future research.

## Results

**Figure 1:**
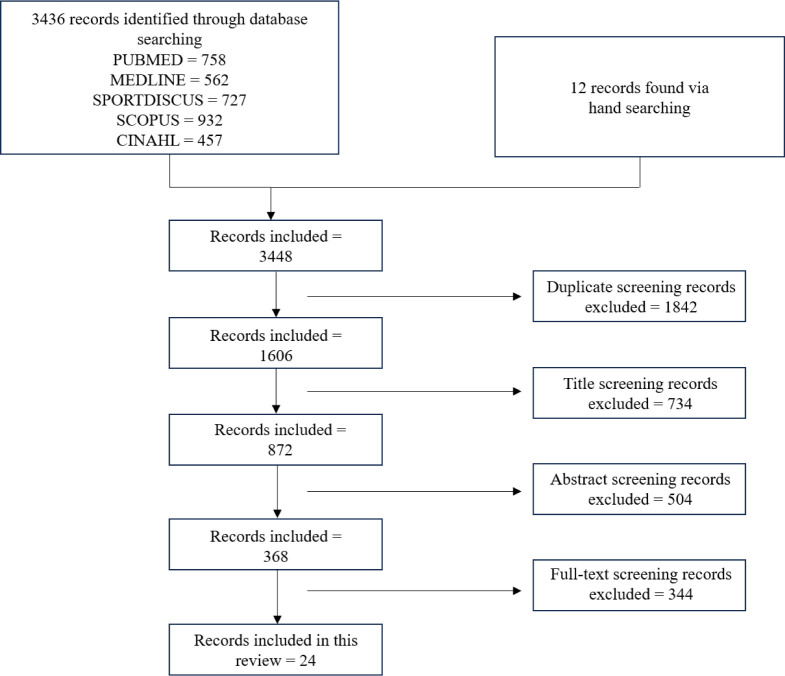
Search and selection process.

The search and study selection processes are presented in Figure 1. After examining the included articles, specific themes emerged which led to the layout of the discussion. The major sections are separated into 1) “the influence of resistance training variables on recovery” and 2) “the influence of programming” (i.e., how the discussed variables were structured and manipulated) on recovery, with sub sections in the former on proximity to failure, volume, and exercise selection, and active recovery (AR), and priming/training cessation also discussed in the latter. Finally, the discussion concludes with limitations and considerations for future research and practical implications.

As shown in Figure 1, a total of 24 articles were reviewed. These articles included a variety of participants, measurements, and outcomes which were extracted from all studies for analysis. A comprehensive description of the extracted results from each reviewed study can be found in Table 1.

**Table 1a:** Summaries of studies included in the review.

Study	Participants	Intervention	Outcomes Measured	Summary of findings
Abadiia et al. (2017)	20 active M	5 x 15, ECC knee flexion. AR (upper STR Tr-3x70%1RM to failure) vs. PR	CK, hamstring STR, DOMS. Pre, 0, 20, 24, 48 h post	AR ↑ slow concentric force. No negative effects in other metrics
Bartolomei et al. (2017)	12 RT M	High VOL: 8x10x70%1RM & high intensity: 8x3x90%1RM in BS	CMJ, iso-kinetic & -metric KE, IMTP, ISO BS, CSA, T:C, IL-6, C-reactive protein, CK, LDH, pre, 30 min, 24, 48, & 72 h post	High-VOL = > performance deficits & MD vs. high intensity
Bartolomei et al. (2019)	25 RT M	High VOL BP (8x10x70%1RM), PR or AR (BP, 5x10x10%1RM) 6-& 30-h post	BP throw, ISO BP & MT pec & triceps, & DOMS 15 min, 24, & 48 h post	BP throw, max ISO force, & pec MT restored 24 h in AR. No diff. DOMS
Bartolomei et al. (2023)	22 RT M	Mixed or block periodisation. Mixed = HYP, POW, STR each session. Block = one per mesocycle 10 wks	BF%, MT, 1RM BS + bench, CMJ + BPT, IBP, ILE, load VOL	Mixed = > FFM, MT &1RM BP. Block = > CMJ
Belcher et al. (2019)	12 well Tr. M	BS, BP, & DL 4x80%1RM to failure	Swell, ROM, DOMS, ACV 70% 1RM, CK, LDH, & cfDNA pre, 0, 24, 48, 72, & 96 h post	ACV < in BS 72 h & BP at 0 h. cfDNA related to ACV all conditions at 0 h
Dourado et al. (2023)	14 un-Tr. young M	Unilateral KE & LP, 8x10x70–90%10RM	Peak torque, CMJ, & MT pre, 0, 24, 48, 72, & 96 h post	LP = < performance & REC rectus femoris muscle edema. VLa REC delayed post KEs
Gonzalez-Badillo et al. (2016)	9 experienced RT M	3x4 vs. 8x80% 1RM in BS & BP	CMJ, V1-Load, T:C, GH, prolactin, IGF-1, CK, HRV & HRC pre, 0, 6, 24, & 48 h post	3 x 4 = < ↓velocity, CMJ, hormonal, MD, HRV & HRC & faster REC
Held et al. (2021)	4 F & 17 M Tr. rowers	Same intensity VBT, 10% VL vs. traditional 1RM Tr., 5 exercises, 4x80%1RMx fail, 8 wks	BS, DL, bench row, & BP 1RM, VO2 max rowing, & REC/stress	VL10 = > BS, row, & BP 1RM & REC & stress 24 + 48 h post
Helland et al. (2020)	8 M & 8 F STR Tr.	1 STR (5RM) & 1 POW (50%5RM) session	BS jump, CMJ, 20m sprint, BS & BP peak POW, e1RM, RPE & PRS pre, 0, 24, & 48 h post	Large NM impairment & > REC times post STR vs. POW session
Howatson et al. (2016)	10 elite track & field athletes	STR (4x5xRPE 17/20) or POW (4x5x30% load) in BS/speed BS, split BS/split-BS jump, push/POW press	MVC, jump height, central activation ratio, & lactate pre, 0, & 24 h post	> NM & metabolic demand post STR impaired max force up to 24 h
Kotikangas et al. (2022)	8 POW + 8 STR athletes & 7 non-athletes	POW (7x6x50%1RM), STR (7x3x3RM), or HYP (5x10x10RM), smith BS	CMJ, T:C, GH, & lactate pre, middle testing, 0, 24 & 48 h post	POW condition = > ↓in POW vs. STR athletes in CMJ. Higher GH in STR athletes vs. non-athletes
Lewis et al. (2022)	16 M & 12 F RT	Pre, REC (4,24, or 48 h), & post REC 4x10RM bicep curls & KE to failure	REC = reps in post REC. Fatigue = reps set to set	REC = no sex diff. Fatigue = < reps in later sets & F > reps bicep curls

**Table 1b:** Summaries of studies included in the review.

Mika et al. (2007)	10 healthy M	3x50%MVC dynamic KE &flexion. PR, stretch, or AR. Post test = ISO KE at 50%MVC	ISO KE, 50%MVC to failure & VLa EMG	Significant ↑in motor unit activation post AR
Moran-Navarro et al. (2017)	10 RT M	3 protocols (sets x reps x [max reps]): 3x5(10), 6x5(10), & 3x10(10), BS & BP	CMJ, MPV V1 & 75% 1RM loads, T:C, GH, & CK at AM + PM, 0, 6, 24, 48, & 72 h post	Failure = > REC of NM function & hormonal homeostasis
Pareja-Blanco et al. (2020)	10 RT active M	Reps (R) per set, max predicted (P): R(P) - 6(12), 12(12), 5(10), 10(10), 4(8), 8(8), 3(6), 6(6), 2(4), & 4(4). 3 sets, BS & BP	CMJ, V1, T:C, GH, prolactin, IGF-1, & CK, -24 h, pre, 0, 6, 24, & 48 h post	Failure = > fatigue, hormonal response, MD, & slower NM REC, especially in high rep sets
Peake et al. (2017b)	9 active M	Single LP & squat, KEs, & walking lunges, 8–12 reps. CWI (10°C, 10 min) or AR (cycling, low intensity, 10 min)	Biopsies pre, 2, 24, & 48 h post - inflammation, cytokines, neurotrophins, & HSP	CWI & AR = similar to minimise inflammatory response
Pritchard et al. (2018)	8 RT M	4-wk STR Tr, then 3.5 or 5.5 days Tr. cessation	T:C, CK, psychological tests, CMJ, IMTP, IBP pre-Tr., final day, & post Tr. cessation	CMJ & IBP ↑over time. CMJ & IBP ↑pre & final day Tr.
Raastad et al. (2000)	10 M STR athletes	100%3RM back & front squat & 6RM KEs or load 70% of that	Isokinetic KEs, ES, & squat jumps pre, 3, 7, 11, 22, 26, 30, & 33 h	All variables REC 3hr post in moderate, 33 h in 100% intensity
Raeder et al. (2016)	14 M & 9 F STR Tr.	6-day STR Tr.: 2x/day, high resistance + max ECC STR, full but mainly lower body	e1RM, MVIC, CMJ, MRJ, RSI, CK, DOMS, PRS, & stress pre & post & 3 days REC	↓in all variables. 3 days, return to baseline in e1RM, CMJ, & MRJ
Thomas et al. (2018)	10 young M	3 x max effort in RT (10x5x80%1RM), jump (10x5 jump squat), & sprinting (15x30 m)	ES KE & fatigue via VAS pre, 0, 24,48, & 72 h post	REC 48 hr jump & 72 h STR & sprint
Travis et al. (2021)	14 M & 2 F PL	6-wk program, 1-wk overreach, either 1-wk step or 3-wk exponential taper	Pre & post ultrasound, biopsies, CMJ, ISO & 1RM BS	Step taper = ↑HYP. Exponential taper = ↑NM performance
Travis et al. (2022)	16 M & 3 F STR Tr. athletes	4-wk PL block. 3 or 5 days of Tr. cessation	Body comp, psychometrics, & BS, BP & DL 1RM pre & post the block & at 3- or 5 days post	No ↓in ISO BS, psychometrics, & body comp. Small ↓in ISO BP post 5, not 3 days cessation
Tsoukos et al. (2018)	17 Tr. M POW + team sport athletes	Low-VOL, POW-type Tr. = 5x4x40%1RM jump squats HYP (3–5x8x75%+1RM),	CMJ, RSI in drop jump, LP max ISO force, & RFD pre, 24, & 48 h post	Low-VOL, POW-type Tr. = > CMJ, RSI, & RFD 24-48 h post
Zourdos et al. (2016)	18 M college PL	POW (3–5x1x80–90%1RM), STR (3x max reps x85%1RM) vs. HYP, STR, POW. BS, BP, & DL	1RM, total Tr. VOL, & T:C pre & post	HYP, POW, STR = > total VOL in BS & BP, > ↑in 1RM BP

Training interventions are expressed as sets x repetitions x load/intensity. ↓ decrease, ↑ increase, ACV average concentric velocity, AR active recovery, BF % body fat percentage, BP bench press, BPT bench press throw, BS back squat, cfDNA cell free DNA, CK creatine kinase, CMJ counter movement jump, CSA cross sectional area, CWI cold water immersion, DL deadlift, DOMS delayed onset muscle soreness, E1RM estimated 1 repetition maximum, ECC eccentric, EMG electromyography, ES electrical stimulation, F female, GH growth hormone, HRC heart rate complexity, HRV heart rate variability, HSP heat shock proteins, HYP hypertrophy, IBP isometric bench press, ILE isometric leg extension, IGF-1 insulin like growth factor 1, IL-6 interleukin 6, IMTP isometric midthigh pull, ISO isometric, KE knee extension, LDH lactate dehydrogenase, LP leg press, M male, MD muscle damage, MPV mean propulsive velocity, MRJ multiple rebound jump, MT muscle thickness, MVC maximal voluntary contraction, NM neuromuscular, PL powerlift-er/ing, POW power, PR passive recovery, PRS perceived recovery scale, Reps repetitions, REC recovery, RFD rate of force development, RM repetition maximum, ROM range of motion, RPE rating of perceived exertion, RSI reactive strength index, RT resistance trained, STR strength, T:C testosterone cortisol ratio, Tr. Train-ed/ing, V1 movement velocity against the load that elicits 1 m/s, VAS visual analogue scale, VBT velocity-based training, VL velocity loss, VLa vastus lateralis, VOL volume

## Discussion

The present narrative review is the first to examine the current evidence regarding the impact of microcycle construction on resistance training recovery. Synthesising and creating practical recommendations from this body of research may help athletes and coaches understand what variables to focus on and how to manipulate them to enhance adaptation. The main findings of our review are that training to failure, greater training volumes, and exercises with higher eccentric torques, especially when they occur at longer muscle lengths, and when more musculature is involved (i.e., lower body exercises) often increase recovery demands. Furthermore, programming strategies can effectively manage fatigue by strategically planning sessions within the microcycle, prioritising easier or less demanding sessions (or even training cessation) to serve as AR before more intense sessions. This approach may reduce fatigue and improve recovery, leading to acute performance enhancements and potentially fostering long-term adaptations.

In the following sections and sub-sections, studies which inform specific approaches to microcycle construction for recovery enhancement are reviewed to inform future coaching practice.

### Influence of Resistance Training Variables on Recovery

There are many factors to consider when designing a resistance training program. When emphasising recovery within a microcycle, proximity to failure, training volume, and exercise selection, all can determine the amount of recovery needed after a training session.

#### Proximity to Failure

Traditionally, it has been recommended that resistance training sets be performed to muscular failure to maximize strength gains and hypertrophy; however, recent meta-analyses have reported no significant differences for muscular strength or hypertrophy (Grgic et al., 2022; Refalo et al., 2023). Importantly, training to failure has also been found to elongate recovery time courses and elicit greater perception of fatigue compared to not training to failure; leading to performance impairments (Vieira et al., 2021). When comparing three sets of eight repetitions to failure with three sets of four repetitions at 80% of the one repetition maximum (1RM) in the squat and the bench press, less fatigue, faster recovery and mean velocities were observed with the latter approach, leading to reduced hormonal response, muscle damage, and less impact on heart rate variability (HRV) and complexity (González-Badillo et al., 2016). Similar results were observed but with three sets of six vs. twelve repetitions at 70% of 1RM (Pareja-Blanco et al., 2016) and across a variety of set configurations (Pareja-Blanco et al., 2020) in the squat and the bench press. Certain variables such as the countermovement jump (CMJ) returned to baseline as soon as six hours post training in the non-failure groups, whereas CMJ performance remained reduced up to 48 h in the failure group.

Notably, in each example, proximity to failure was manipulated with a static number of sets and loads, leading to lower volume in the non-failure comparisons, warranting further volume-equated investigation. However, in such subsequent comparisons when volume was equated, training to failure still increased recovery demands. For example, metabolic markers of fatigue and low, medium, and high load strength performance required 24–48 h longer to return to baseline in a group completing three sets of 10 repetitions to failure compared to six sets of five repetitions with the same load (Morán-Navarro et al., 2017). Therefore, even when volume is equated, a closer proximity to failure has an independent impact, elongating the time course of recovery.

Another common way to prescribe resistance training loads and volume is via velocity-based training (VBT). Loads can be prescribed by targeting a specific mean concentric velocity on initial repetitions in a set, and the subsequent set-volume can then be regulated based on neuromuscular fatigue by stopping a set after a repetition produces a certain amount of velocity loss as the set approaches failure (expressed as a percentage). The approach of VBT may be favoured as it offers a more individualised approach which can help account for the variability seen when using a traditional percentage-based approach (Cooke et al., 2019). Confirming the prior research comparing failure to non-failure training in a VBT model, a 10% velocity loss led to greater back squat, prone row, and bench press 1RM improvements in addition to greater recovery and improved stress levels compared to traditional sets to failure with 80% 1RM in one study (Held et al., 2021). When examining 15 vs. 30% velocity loss in the leg press and leg extensions, no statistically significant differences between increases in strength or muscle thickness were observed (Andersen et al., 2021). However, a recent review with meta-analysis reported that when sets and relative intensity were equated, velocity loss thresholds ≤25% were superior for promoting strength potentially due to minimising acute neuromuscular fatigue while maximising chronic neuromuscular adaptation. Conversely, velocity loss thresholds > 20–25% were superior for promoting hypertrophy by accumulating greater relative volume (Hickmott et al., 2022). Importantly, upon further analysis, it seems that if velocity losses >20% are compared when set volume and relative loads are equated, differences in the volume load have little to no additional impact on muscle hypertrophy. Rather, other factors such as neuromuscular fatigue may preside over the influence of proximity to failure on muscle hypertrophy for different velocity loss thresholds (Refalo et al., 2023). While the use of first/submaximal repetition velocity and velocity loss to predict 1RM and prescribe training have been proposed (García-Ramos et al., 2018), evidence suggests these methods can be highly variable and inaccurate (Haischer et al., 2023; Macarilla et al., 2022); thus, if chosen, should be viewed as a supplementary piece of data used in conjunction with additional autoregulatory or individualisation strategies to help in the decision making process. Therefore, given the totality of data on VBT, while higher thresholds may produce slightly more hypertrophy on average (Jukic et al., 2023a), it is important to consider the individual response to different velocity loss thresholds as proximity to failure can vary substantially (Jukic et al., 2023b). Furthermore, when considering their implementation within a microcycle, a session using higher velocity loss thresholds may potentially result in greater fatigue and recovery times, especially when those thresholds result in training closer to failure.

While performance, hormonal response, and more objective measures give important insight into recovery, perceptual responses must be considered as they also influence how an individual approaches a training session. Specifically, when comparing sets to failure vs. not to failure across four sets in the back squat, training to failure resulted in more repetitions during the first set and non-failure training resulted in more repetitions on the last set with total repetitions across all sets being similar (Santos et al., 2018). Despite total repetitions being similar between groups, velocity across all repetitions in the non-failure condition group was faster and self-reported exertion and discomfort were greater under the failure condition. Therefore, with similar performance outcomes and a higher RPE and discomfort reported, it is apparent that training to failure imposes an extra perceptual recovery demand which should be considered when designing training micro- and mesocycles.

Overall, training to failure can increase recovery times, potentially negatively impacting subsequent performance on high priority sessions. Such an impact warrants careful consideration of training stress allocation when programming. However, training to failure may have a time and place if implemented with caution. Specifically, if sufficient time is given between sessions involving the same muscle groups, adequate recovery may be achieved, minimising any potential negative consequences. Application of failure training may be more feasible in isolation movements involving less musculature (i.e., leg extension vs. leg press) (Dourado et al., 2023), machine-based exercises as opposed to high-skill, demanding barbell movements (Haff, 2000; Saeterbakken and Fimland, 2013), or that emphasise shorter muscle lengths (Nosaka and Newton, 2002), as each may have lower recovery demands. Additionally, one could perform only the last set of an exercise for a given muscle group at the end of the session to failure so as not to have fatigue bleed into subsequent exercises of that session. If applied appropriately, training to failure in such a manner could not only yield an increased stimulus, but may help individuals accurately gauge their RPE in subsequent training by better anchoring the point of muscular failure. However, further research is required to determine exactly how far from failure one can be to balance stimulus and stress, for which movements, and in what time course relative to high priority sessions to maximise such outcomes.

#### Volume

Training volume also plays a significant role in training outcomes, particularly for hypertrophy (Currier et al., 2023); however, more may not always be better and may come at a cost. When comparing 12–20 sets per muscle group per week to 20+, there were no significant differences in muscle hypertrophy for most muscle groups (Baz-Valle et al., 2022). Practically, if similar results can be obtained with roughly half of the work, one must consider the potentially greater fatigue accumulation from higher volume sessions and whether the, at best, marginal improvements in adaptation are worth the cost of an increased recovery time course between sessions and any negative impact on subsequent performance.

Notably, high volume training is not performed in a vacuum. Its effects interact with proximity to failure. For example, when volume is equal between conditions, similar outcomes in hypertrophy are typically seen with strength largely moderated by the load (i.e., higher loads leading to better strength improvements) (Andersen et al., 2021; Carvalho et al., 2022). Indeed, while a reasonably strong (albeit non-linear) dose response between higher set volumes and hypertrophy exists (Schoenfeld and Grgic, 2018), the relationship between higher set volumes and maximal strength is trivial to small (Ralston et al., 2017).

When the goal is increasing maximal strength, given the relatively minor impact of volume on adaptation, the impact of higher volumes on recovery should be considered. For example, when comparing an acute bout of high volume, moderate load with short rest training to moderate volume, high load with longer rest training, various measures of muscular strength and power decreased significantly more and for a longer period of time after such high-volume training (Bartolomei et al., 2017). Similarly, as discussed, high volume, high velocity loss training results in greater neuromuscular fatigue and recovery times compared to high load, high RPE training with various set configurations (Pareja-Blanco et al., 2019, 2020).

Overall, within a microcycle, specific consideration should be given to proper volume allocation as greater volumes within a session may impose greater recovery demands. Specifically, if higher volume sessions are to be introduced, proper placement of these sessions must be considered as inadequate recovery times may impair performance in subsequent sessions. A wide range of 10–20 sets per muscle group per week is associated with superior hypertrophy (Baz-Valle et al., 2022; Schoenfeld and Grgic, 2018) and a wide, albeit lower, range of 5+ sets per movement per week is associated with superior strength gains (Ralston et al., 2017), demonstrating that higher volumes may have utility, on average. However, the individual response to higher or more moderate volume is notable (Damas et al., 2019). Thus, like training to failure, high volume training should be implemented with caution and purposefully. Specifically, higher volumes could be implemented for a certain muscle group or movement in a “specialisation phase” while volume is brought to lower or “maintenance” levels for other muscle groups or movements of interest. Such a strategy may prevent the accrual of excess fatigue while increasing the stimulus on the target muscle group/movement. Furthermore, to avoid exceeding individual volume tolerances, it may be wise to start on the lower end of volume prescriptions, assess recovery, then if needed gradually increase volume on specific days of the microcycle while accounting for the likely increased recovery time course impacting subsequent days. Finally, the additive effects of higher volume and closer proximity to failure should be considered, as this relationship requires further empirical exploration, because their combination may compound fatigue and recovery demands if implemented inappropriately.

#### Exercise Selection

While the relationship among volume, fatigue, and adaptation has been examined as total working sets performed per week in recent meta- analyses (Baz-Valle et al., 2022; Ralston et al., 2017; Schoenfeld et al., 2019), *how* that volume is performed (i.e., how frequently it is distributed within the week and with what exercises) may moderate this relationship. Indeed, in four recent meta-analyses, three assessing the impact of how frequently exercises are performed in a week on maximal strength gains (Cuthbert et al., 2021; Grgic et al., 2018; Ralston et al., 2018) and one examining the impact of how frequently muscle groups are trained in a week on hypertrophy (Grgic et al., 2019), there was a positive relationship between higher frequency training and adaptation, but only when higher frequency training led to higher volumes, with the effect diminishing or disappearing in volume-equated sub analyses. Indeed, the authors of those analyses suggest that training frequency can be looked at as a tool to allocate weekly training volume in an appropriate manner, facilitating more efficient, high-volume training by allowing inter-session recovery (Grgic et al., 2019; Ralston et al., 2018; Schoenfeld et al., 2019). Thus, frequency manipulation may be used to decrease fatigue accumulation and allow for adequate recovery throughout the microcycle. However, it is important to consider the type of exercise used to accumulate volume as this may influence the time required to recover.

Specifically, when comparing lower versus upper body exercises, greater recovery times are needed for the lower body (48–72 hours) compared to 24 h or less for the upper body (Bartolomei et al., 2017; Belcher et al., 2019; Lewis et al., 2022; Raeder et al., 2016). However, even within the lower body, differences between exercises can be observed as greater impairment of functional performance and delayed recovery of muscle edema was reported in the leg press compared to knee extensions (Dourado et al., 2023). This suggests that multi-joint movements may require additional recovery times perhaps due to the greater amount of musculature involved, the subsequently higher absolute loads used, and greater coordination demands (Haff, 2000; Saeterbakken and Fimland, 2013). The degree to which exercise selection impacts recovery may vary depending on how the microcycle is organised (i.e., upper/lower split vs. full body, etc.), how other training variables are manipulated, and the goals of the individual as these aspects operate interdependently, but nonetheless, exercise selection warrants specific consideration.

Another way to categorise movements is by the type of contraction and the portion of the length-tension relationship in which they primarily operate. Notably, when comparing full to partial range of motion (ROM) training, while full ROM training produces greater strength and hypertrophy than partial ROM training at shorter muscle lengths, partial ROM training at longer muscle lengths may produce similar if not slightly superior muscular adaptations to full ROM training (Wolf et al., 2023). With that said, higher volumes with exercises that produce high versus low eccentric torques at long versus short muscle lengths resulted in more severe exercise induced muscle damage (Nosaka and Newton, 2002). Thus, while such exercises, contraction modes (i.e., eccentric training) or performance techniques (i.e., purposeful long-muscle length partial ROM training) may enhance the stimulus of training, they also may increase fatigue, requiring coaches and athletes to consider their placement in a microcycle and monitor and assess recovery.

To conclude, exercise characteristics such as where the highest torque occurs on the length-tension relationship of the trained musculature, a contraction mode, total musculature trained, and ROM influence recovery, and thus, should be considered when constructing a microcycle. Specifically, exercises that are lower body focused, multi-joint, more complex, recruit greater musculature, emphasise the lengthened position, and/or emphasise the eccentric portion of the movement should be strategically placed in a microcycle as greater recovery times may be needed following their performance. When implemented, the volume allocated to such exercises and their frequency can be manipulated to alter the time course of recovery; however, the specific application of such programming decisions will heavily depend on the goals of the individual and the logistical demands of their current training, but nonetheless, warrants consideration.

### Influence of Programming on Recovery

After considering how these variables may influence recovery individually, it is important to examine how they interact and subsequently, how this may influence the program design. However, such a discussion requires the proper use of terminology: programming versus periodisation (Hammert et al., 2021; Hornsby et al., 2020). Periodisation can be viewed as having a particular focus (i.e., strength or hypertrophy) for a phase of training (>6 weeks) that fits within the larger overall design (macrocycle), whereas programming involves manipulation of training variables within these phases that in turn, emphasise maximising the desired outcomes.

When comparing the magnitude of strength gains and hypertrophy between volume-equated periodised and non-periodised strength programs, periodised training led to significantly greater strength gains than non-periodised with no significant differences in hypertrophy, while undulating models produced greater strength gains than linear models in trained individuals (Moesgaard et al., 2022). However, undulating models are arguably programming not periodisation strategies and thus, it is important to examine what specific programming changes may lead to such results to further understand a program design. Consequently, one proposed method of undulating programming is to designate a specific focus of training on each day such as strength, hypertrophy, and power (i.e., DUP). When comparing two DUP models with a weekly order of either HSP or HPS across six weeks of training, there were greater training volumes produced in the squat and the bench press and greater increases in the 1RM bench press in favour of HPS (Zourdos et al., 2016). Likewise, a similar DUP approach produced greater improvements in the bench press 1RM and pectoral muscle thickness compared to block periodisation (Bartolomei et al., 2023). Therefore, the structure and order of each training session within a microcycle may have an influence on recovery, and performance in subsequent training sessions, which summate to produce better outcomes over the course of a full mesocycle. These acute programming benefits may be due to greater neuromuscular impairments and recovery times seen after a strength compared to a power session (Helland et al., 2020) and thus, it is speculated that by placing an “easier” day in between two “harder” days as opposed to having two “hard” days in a row, individuals may be able to perform better throughout the week. However, outcomes may vary for individuals based on their training background which can also influence acute responses, thus, warranting sport specific and individual-specific consideration (Kotikangas et al., 2022).

When comparing the effects on strength versus power style back squats on neuromuscular fatigue, heavy loads led to significant reductions in power, maximal voluntary isometric contraction, the rate of force development, and evoked peak twitch force while light sessions resulted in no change in power production during, and the least number of decreases in performance post-training (Howatson et al., 2016). Essentially, due to the low fatigue nature of power-type training, individuals may experience better recovery between sessions in a modified DUP model. With that said, these mechanisms were not the specific aim of the aforementioned study, so the underlying mechanistic rationale is yet unclear. Ultimately, regardless of the mechanism, it seems the placement and difficulty level of specific exercises or sessions can influence recovery and performance which should be considered when constructing a microcycle.

DUP can be modified to elicit better performance in harder/priority days, while still making easy days purposeful, and productive. However, knowing exactly what easy days should consist of, and how many easy days relative to hard days should be performed, is yet unexplored. Another option besides implementing power days for this purpose is light cardio aerobic sessions, possibly prompting AR better than actual training. However, it is also possible that a priming effect that enhances subsequent day power or strength performance could occur with an appropriately structured light resistance training power day, or, finally, full training cessation could possibly be the best option in some instances to fully maximise recovery prior to a very challenging session.

#### Active Recovery

Low fatiguing power sessions may act as a form of AR defined as any form of exercise as a method to improve recovery (Barnett, 2006). Typically, this exercise is of lower volume, intensity, and duration and is performed during the recovery bouts of exercise (Spierer et al., 2004) or during the recovery phase after a training session (Mika et al., 2007). The proposed mechanisms to enhance recovery are reductions in muscle edema, enhanced muscle fibre generation, and a decrease in the inflammatory response from high-demand exercise sessions (Clarkson et al., 1992; Clarkson and Sayers, 1999; Peake et al., 2017a). AR is an effective technique for improving recovery after physical exercise (Dupuy et al., 2018); however, few studies have examined the effects of AR after resistance training. Compared to cold water immersion (CWI), AR consisting of low intensity cycling had similar effects on the inflammatory response following high-volume lower body resistance training (Peake et al., 2017b). Consequently, if recovery was similar, then opting for AR may be preferred due to ease of implementation, accessibility, and to avoid the potential negative effects that repeated CWI may have on muscle hypertrophy (Fyfe et al., 2019; Roberts et al., 2015).

Low-volume power-type sessions are a common way to prescribe AR in resistance training. Specifically, when an upper body exercise session was performed the day after a lower-body workout, recovery rates of strength performance were improved compared with passive recovery (PR), with positive outcomes attributed to changes in the microvascular blood flow and increased concentrations of anabolic hormones after exercise (Abaidia et al., 2017). Importantly, there were also no observed negative effects due to the inclusion of an upper body session after a damaging lower body session, indicating the potential utility of placing sessions which train different muscle groups in close proximity to one another as means of AR. However, it is important to monitor individual responses as total body and/or mental fatigue may linger, potentially impairing subsequent performance (Kotikangas et al., 2022). Another method of implementing AR, rather than including a different exercise or training a different muscle group, is training the same exercise, but far lighter. For example, light bench press AR sessions performed 6 and 30 h after a high-volume exercise protocol accelerated reductions in pectoralis major muscle swelling compared to a PR group whose pectoralis major muscle thickness was still significantly increased from baseline 48 h post-training (Bartolomei et al., 2019). While these results show promise, and could plausibly enhance adaptation longitudinally, practically, the study protocol of 5 sets of 10 repetitions with 10% of 1RM may not be feasible for certain exercises and other ecologically valid options should be explored.

Overall, AR could take the form of training opposing muscle groups on back-to-back days, or could consist of light aerobic cardio sessions or low-volume power-type resistance training, all of which may enhance recovery compared to PR. When choosing a modality that involves similar muscle groups or exercise patterns, the proposed mechanism of recovery enhancement should take into account an improved blood flow to the designated area. However, appropriate volume and intensity of AR are crucial, as improper manipulation of training variables may lead to exercise being too taxing, and counterproductive (Zarrouk et al., 2011). As the exact volume, intensity, and frequency of AR are not yet well understood, further research is needed to develop specific guidelines for application.

#### Is It Priming, Recovery or Simply Training Cessation?

When exploring the effects of AR, the question to whether results are due to dissipation of fatigue or a potential “priming” effect, or both, warrant consideration. While, the idea of resistance training performed 24–48 h before a competition “priming” performance is proposed in textbooks, research on the efficacy of this concept is limited (Mujika, 2009; Raastad and Hallen, 2000). Such proposed benefits have been attributed to post activation performance enhancement (PAPE), the increase in muscular contractile capacity following a high intensity voluntary contraction (Wyland et al., 2015). Thus, the rationale of what causes the outcome of “priming” (i.e., an acute performance enhancement) is that a low volume RT session may result in not only improved recovery, but also a short-term supercompensation of explosive strength performance (Bishop et al., 2008). However, such speculation is relatively unexplored, and further investigation is needed. With that said some evidence does support this theory, as Tsoukos and colleagues (2018) examined the delayed effects of a low-volume, power-type training priming session on explosive performance 24 and 48 h after priming. This priming session, consisting of jump squats of 5 sets of 4 repetitions with 40% of 1RM, led to the greatest increase in explosive muscle performance 24 h post-session. While these results are intriguing, jump squats are not typically programmed for those with the goal of increasing hypertrophy and strength. Thus, future work should see if priming with greater specificity confers similar or improved benefits, with increased ecological validity and coach/athlete buy-in.

To determine whether AR has utility primarily via recovery and not priming, however, it is important to examine whether similar outcomes can be observed by strategically decreasing training demands via tapering or training cessation. When comparing a step vs. exponential taper, enhanced skeletal muscle adaptations and neuromuscular performance have been observed (Travis et al., 2021). Additionally, Pritchard and colleagues (2018) demonstrated that taking either 3.5 or 5.5 days off resulted in significant improvements in CMJ height and isometric bench press relative peak force with no significant differences between the two periods. However, maximal lower-body strength was preserved, but not enhanced, during 3 and 5 days of training cessation while upper body strength slightly decreased after 5 days of training cessation in another study (Travis et al., 2022). Therefore, it is possible that the positive effects of training cessation on maximal strength expression only last so long, are likely due to short term decreases in neuromuscular fatigue and improved recovery, and suggest that a gradual decrease in volume or a short-term cessation of training may be a strategy to consider when constructing micro- and mesocycles. However, it is possible that such strategies might be further improved if combined with priming.

Consequently, implementing AR within microcycles in a purposeful manner may allow for improved recovery and subsequent performance increases as a result of a potential PAPE priming effect. However, the specific window of time, exercises, and prescription guidelines for combining priming AR sessions and training cessation or tapers requires further investigation. However, theoretical approaches based on existing data can be proposed. For example, at the end of a 4-week mesocycle, a taper could commence in the fourth week, with a priming session on the second to the last day of the week, followed by 1RM testing to be performed on the following day. Similarly, instead of a taper, in the fourth week training could be reduced from five days per week to two, with the first three days being skipped, and the last two consisting of a priming day and a 1RM testing session on the following day. However, these suggestions are speculative, as it is unclear, based on the current research, how to manage such programming strategies in a systematic manner.

## Limitations and Considerations for Future Research

There are several limitations that should be considered when interpreting or applying the herein discussed concepts. While a systematic approach was adopted to select the current studies, this review is in a narrative format. The narrative style was chosen to provide a more descriptive approach of the literature and propose more practical implications, but the current literature is not yet at a confidently prescriptive stage. Ultimately, this review focuses on the ecological validity of the proposed topic and how individuals may utilise this information in their own training practices. The studies included vary in terms of the population, training protocols, and measurement techniques which may influence the generalisability of the findings. Due to studies being conducted in controlled laboratory settings, extrapolating these findings to real world training settings requires careful consideration as numerous variables may influence outcomes. Individual responses to training outcomes are also widely documented (Bamman et al., 2007; Hubal et al., 2005); therefore, any application from this discussion should be considered within the context of an individualised approach. Practitioners are advised to monitor objective and subjective individual athlete data to determine the success or lack thereof of any novel recovery strategy, and such monitoring should dictate subsequent changes to training as appropriate.

Due to the multifaceted nature of recovery, it is often difficult to determine if a specific variable was indeed what caused a result. For example, if studies did not control or monitor sleep, nutrition, hydration or stress, these variables may have influenced outcomes (Bartholomew et al., 2008; Beck et al., 2015; Bird, 2013; Judelson et al., 2007). Future research should attempt to account for these variables in order to provide an additional context and understanding when interpreting results. Limited research exists examining the long-term effects of emphasising recovery when constructing a microcycle. Of the research that exists, recovery is typically not directly assessed but inferred by performance (i.e., repetitions completed, muscle thickness, 1RM, etc.), making the determination of a mechanism or mechanisms impossible. In the future, research specifically examining recovery and performance over longer duration, while assessing potential mechanistic causes to delineate the effect of recovery versus priming, is suggested to further enhance our understanding of how these variables influence microcycle construction.

## Conclusions

While training to failure may lengthen recovery periods, if performed cautiously, it can provide utility. For example, with sufficient time between training sessions which engage the same muscle groups, adequate recovery may occur. Training to failure may also be more practical when applied to machine-based exercises rather than high-skill barbell movements, isolation exercises involving less musculature, or exercises that emphasise shorter muscle lengths to reduce recovery demands. Additional consideration should also be given to volume allocation as greater training volume within a session also increases recovery time. Volume may be most easily quantified as the number of sets per muscle group and/or a movement pattern per week, and on average results are optimized in the range of 10–20 sets per muscle group for hypertrophy and a smaller but wide range of 5+ sets per movement for strength. However, individual responsiveness to higher or more moderate volume is noteworthy. Therefore, like training to failure, high volume training should be undertaken with care and purposefully planned. For example, higher volumes could be utilised for a certain muscle group or movement in a “specialisation phase”, while volume could be reduced to lower or “maintenance” levels for other muscle groups or movements. This approach could increase the stimulus to the targeted muscle group/movement while preventing the accumulation of undue fatigue. Practically, these concepts can be applied with a DUP format as well, as it may be wise to start on the lower end of volume prescriptions, evaluate recovery, and then, if necessary, gradually increase volume on particular days of the microcycle where the increased recovery time will not unduly impact subsequent days. Importantly, the additive effect of higher volume and closer proximity to failure should be accounted for as this combination may compound fatigue and recovery demands if implemented inappropriately. However, further research is required to enhance the understanding of their interaction.

Frequency may be viewed as a tool for disseminating weekly training volume to manage fatigue and allow for adequate recovery during the microcycle. For example, instead of designating a large amount of volume in one session for a specific muscle group or movement, it can be spread across two or three sessions. Additional consideration should be given to exercise characteristics as those that target the lower body, involve multiple joints, recruit greater musculature, emphasise the lengthened position, and/or the eccentric portion of the movement may require greater recovery times. Thus, greater volume and/or exercises with such characteristics placed further away from sessions of priority may lead to less fatigue accumulation allowing for maximal performance on that day. A DUP format whereby low-volume power-type or aerobic sessions placed between more demanding sessions may improve performance in such priority days. This may be viewed as a form of AR and/or synergistically invoke a possible priming effect that improves subsequent day power or strength performance. Practically, training opposing muscle groups or selecting low-fatiguing modalities that involve similar muscle groups or exercise patterns on consecutive days may improve recovery via an enhanced blood flow. For example, a light cycling or an upper body session prior to a lower body session would likely not result in high enough fatigue levels to impair performance on the subsequent day. Furthermore, tapering and cessation of training may be used in conjunction with priming sessions to allow for maximal performance on testing sessions. For instance, a taper or reduction in training may begin on the last week of a mesocycle with a priming session placed one or two days prior to the day of testing. However, the exact volume, intensity, frequency, and the specific time window of AR and implementation of such programming strategies are not well understood, therefore, further research is necessary to provide specific guidelines for application.
